# “Like another human being in the room”: a community case study of smart speakers to reduce loneliness in the oldest-old

**DOI:** 10.3389/fpsyg.2024.1320555

**Published:** 2024-04-22

**Authors:** Arlene Astell, David Clayton

**Affiliations:** ^1^Psychology Department, Northumbria University, Newcastle Upon Tyne, United Kingdom; ^2^School of Psychology and Clinical Language Sciences, University of Reading, Reading, United Kingdom; ^3^Department of Occupational Therapy and Occupational Sciences, University of Toronto, Toronto, ON, Canada; ^4^Department of Psychiatry, University of Toronto, Toronto, ON, Canada; ^5^The KITE Research Institute, Toronto Rehabilitation Institute, University Health Network, Toronto, ON, Canada; ^6^Department of Cardiovascular Sciences, University of Leicester, Leicester, United Kingdom

**Keywords:** social isolation, loneliness, oldest-old people, smart speaker, agency, presence

## Abstract

This community case study examined the potential benefits of smart speakers to tackle loneliness in the oldest old adults living in supported accommodation. The program was established as a collaboration between the supported accommodation provider and a technology company to explore the feasibility of smart speakers to alleviate resident loneliness. Loneliness in later life often accompanies a shrinking social circle, loss of a spouse or increased disability. People aged 85 years of age and over are increasingly likely to experience these life events, leading to an increased risk of social isolation and loneliness. Five older people, mean age 90 years of age, who resided in supported accommodation, were given a smart speaker for 8 weeks to examine their experience with the voice assistant. The experiences of the five older adults are explored as case studies, with each person interviewed both before and after receiving the smart speaker. All five valued their smart speaker, recognised its potential for tackling loneliness, and wanted to keep it. The three most lonely individuals reported that their smart speaker made them feel less lonely and isolated through two mechanisms: (i) creating a presence and (ii) having some control over their situation. Although only a small study, these experiences suggest providing smart speakers for lonely and isolated oldest-old people, could be one way to help combat loneliness in community settings.

## The problem: loneliness among the oldest-old

The number of “oldest old” (Suzman et al., 1992) people in the UK is rising because of improving standards of living and healthcare. A distinction may be drawn between those older people in the third age and fourth age (Laslett, 1991; Higgs and Gilleard, 2015); the difference is between an older, healthy and productive life in the third age, and illness, disability and need for care and support in the fourth age. Although not determined by chronological age, the third age and fourth age have been associated with the “young-old” (60–70) and “old-old” (after 70) respectively (Baltes, 1998). However, the term oldest old has been variously applied to people over 75 years (Poon and Cohen-Mansfield, 2011), 80 years (Gjonca et al., 2010), and 90 years of age (Dini and Goldring, 2008), with the most widely used definition as 85 years of age and over (Key and Culliney, 2018), which is the population the current study is concerned with.

In the United States, the oldest old are the fastest growing segment of the over 65 population, with an estimated 6.6 million adults over age 85 in 2019 (Administration for Community Living, 2021). In the UK, the Office of National Statistics (2018) found that the fastest growing older group are the over 85 s; predicting they will constitute 4% of the population by 2041. More recently, the United Nations, Department of Economic and Social Affairs, Population Division (2020) highlighted that the number of over 65 s worldwide will more than double by 2050 after previously predicting there will be 25 million centenarians by 2,100 (United Nations, 2015).

As the number of oldest old has grown, so has the research into this population, with a particular focus on health-related topics (Lund and Wang, 2020). This focus is unsurprising as, for example, hospital and community care costs in the UK, are estimated to be up to three times higher for people over 85 years than those aged 65–74 years (Cracknell, 2010). Factors that influence mental in later life well-being have been studied through analysis of longitudinal datasets and suggest increased vulnerability to depression in the oldest old (Lee et al., 2020) and increased risk of loneliness (Victor et al., 2008). In the UK Newcastle 85+ study, for example, both mild and severe depression (using GDS; Sheikh and Yesavage, 1986) increased the risk of loneliness in the oldest old (Brittain et al., 2017). At entry to the study, 55% of respondents over 85 years of age reported they were often or always alone, with women spending more time alone than men. A recent systematic review of the longitudinal risk factors for loneliness in older adults found several consistent associations with loneliness including depression, partner loss and poor health (Dahlberg et al., 2022).

Recent widowhood and disability accord with the five ways older adults experience loneliness proposed by Clayton (2018): (i) A feeling of loss or sadness triggered in the moment (e.g., by symbolic events such as sitting down to watch TV and realizing a life partner is not there); (ii) a change in identity (e.g., loss of former roles, moving away from familiar community, becoming a caregiver for a family member); (iii) a loss of intimacy and grief (e.g., loss of close personal relationship, often after many years); (iv) reduced choice and control (e.g., over where to live, where to go out); or (v) poor health and disadvantage (e.g., physical, and mental deterioration, lack of mobility), all factors mentioned in studies of the oldest-old.

Additionally, the UK Understanding Society Wave 3 dataset identified the oldest old at increased risk of social exclusion compared with adults aged 65–84 years of age. In this analysis, social exclusion was defined as exclusion from services and exclusion from social relations (Key and Culliney, 2018), both of which contribute to loneliness and social isolation. From a research perspective, loneliness and social isolation are considered as two separate but related constructs. Loneliness is the *subjective* experience of being alone and is described as a mismatch between the quantity and quality of our social relationships, and those that we want to have (Perlman and Peplau, 1981). While loneliness is a subjective experience, social isolation is the *objective* social state of having limited social contacts or interactions with other people. Methodologically, social isolation can be measured by counting the number of contacts a person has (National Seniors Council, 2017), which influences an individual’s experience of loneliness (Weiss, 1973).

Traditional approaches to tackling loneliness favour befriending and social groups but the Covid-19 pandemic, with its enforced social isolation and movement of many vital services online, highlighted the potential and importance of digital technology (Clayton and Astell, 2022). However, the oldest-old adults may be disproportionately excluded from accessing internet-based resources (Friemel, 2016). Voice Activated Technology, including smart speakers and voice assistants, is considered more accessible than many other digital devices (Pyae and Joelsson, 2018). Voice provides more natural interactions, ease of use and control for the user (Pradhan et al., 2018). In their study of 12 adults over 65 years of age using Google Home Hub for 16 weeks, Kim and Choudhury (2021) reported that the participants became more confident using voice assistants and developed “digital relationships”. Other studies have found that users may form an attachment with voice assistants as they become embedded into their everyday lives (Lopatovska and Williams, 2018; Ramadan et al., 2021). In one study, 62% of Smart Speaker users felt less isolated (Argenti, 2018), with voice assistants offering a form of companionship (O’Brien et al., 2020). Companionship is also experienced by older adult users of voice assistants (Corbett et al., 2021). The experience of companionship may be due to anthropomorphism where the voice assistants are attributed humanlike traits (Liu, 2023). Additionally, anthropomorphism, appears to play a role in reducing loneliness (Jones et al., 2021). Together, these findings suggest the potential for voice assistants to impact loneliness amongst oldest old adults.

### Background and rationale

This community case study was initiated by a provider of supported accommodation in the UK collaborating with a local technology company. Supported accommodation, also referred to as assisted living or sheltered accommodation in the UK, is a type of housing where individuals have their own rooms or apartments, with access to shared facilities including catering, social space and activities, plus on-site support staff. The location for this project is home to 25 residents and provides on-site support between 9 am and 6 pm. The two companies were interested in offering their older residents the opportunity to try a smart speaker – Google Home – to explore the impact on loneliness.

The technology company is a human tech agency that utilises digital equipment and data to create engagement between people to improve quality of life. The company saw the potential for voice technology to help one of their clients, a provider of supported accommodation for older people, to further their mission to alleviate loneliness amongst their residents. Their residents are often the oldest old who have experienced bereavement, which can lead them to experience loneliness. This pilot aimed to meet the human need to talk to others by introducing personal smart speakers into the lives of a small number of older people supported by the provider and seeing if talking to the assistant helped alleviate loneliness. The academic partner was invited to join the collaboration to provide the research background and context to the study and co-produce a means to evaluate the pilot study that could be conducted in the community setting. The rationale for the study was to determine if adults over 85 years of age experience benefits found in previous studies relating to companionship and social isolation.

### Methodology

The #VoiceForLoneliness project^1^ was established in and undertaken in a community setting as a partnership between different stakeholders. The participants lived in the same supported accommodation and were interviewed in their own homes. We adopted the format of a community case study after Smith et al. (2016) which comprises: description and reflection on an intervention (in this case, providing smart speakers to residents), within a community setting (the supported accommodation) to improve the health or functioning of an individual (reduced loneliness). The intention was to document the experience of older adults using smart speakers to cope with loneliness in supported accommodation to contribute to future evidence-based practice.

### Participation in the study

Five residents were selected by the house manager of the supported living accommodation where the initial pilot was due to take place. The inclusion criteria were to be over 85 years of age, have no diagnosis of dementia or current mental illness, able to speak, read, write, and understand English (with the use of corrective devices, e.g., glasses, hearing aid, if necessary). Two males and three females, all Caucasian, aged between 87 and 95 years of age, with a mean age of 90 years of age, took part. They all lived alone and were residents in the same supported living accommodation. All participants had experienced bereavement and the loss of their spouse. In some cases, this prompted their move into supported housing.

### Ethical approval

The study received approval from the University of Reading Research Ethics Committee (2019-089-AA). Each participant was provided with information about the study and invited to discuss it with members of their family and the research team. Once their questions were answered, those who were interested were asked to provide written consent. Participants were advised that the smart speaker would record their voices but that they could withdraw from the study at any time.

### Procedure

A pre-smart speaker semi-structured interview was conducted individually in each participant’s room or apartment before installing the smart speaker. The interview included collecting data on age, gender, current mobile/smartphone use, current computer use, current pastimes, and asking a single-item loneliness question taken from the Centre for Epidemiologic Studies Depression Scale (CES-D: Radloff, 1977). The CES-D loneliness question asks about loneliness during the past week reported as “rarely/none of the time”, “some or little of the time”, “occasionally or moderate amount of time”, and “most or all of the time”, scored from 0–3.

After the interview, the smart speaker was installed (Google Home smart speaker; current cost for comparison £49.00/$45.00) by the technology company, and each participant received instructions plus a demonstration of how to use it. The demonstration included how to activate the assistant, how to adjust the volume, and different types of questions it could be asked, such as what the weather will be or how to start playing a game. The residents were encouraged to practice asking questions, so they became familiar with the correct format. A “cheat sheet” including all of the above instructions was also given to each participant. Each demonstration was tailored to the support needs of each participant to ensure they were comfortable and confident using the device.

Each participant had the smart speaker for their personal use for eight weeks. After this time, a second interview was completed, again using a semi-structured approach. This interview covered the participant’s thoughts on the smart speaker, the impact of the device on their life, what they used the device for, daily routine with the device, thoughts on voice technology, other features they would like, whether they would keep it, and how they would feel without it. Both pre-and post-interviews were video recorded using an HD video camera and transcribed for analysis.

### Data treatment

Data from the pre/post-interviews data were combined to form 5 individual case reports of a wider community study to gain an in-depth understanding of a small number of cases located in real-world contexts (Yin, 2009). Using the pre-and post-interview data, experiences could be compared and contrasted between the five participants using the smart speakers. This allowed different aspects of the experiences to be revealed and understood, deconstructing, and reconstructing the phenomenon (Baxter and Jack, 2008). In the results that follow, pseudonyms are used for all participant’s case stories. The presentation of stories is in a narrative form below, giving agency to “cases” rather than simply variables (Bazeley and Jackson, 2013).

### Results

The five participants were all over 85 years of age, putting them in the category of the oldest old (Table 1). Three out of the five participants reported feeling lonely some or all of the time. The other two had previously experienced loneliness but did not report it being a current problem during the first interview. All used a mobile phone and all but one owned a tablet or computer (Table 1). None had previously used or owned a voice assistant.

**Table 1:** Participant characteristics before using the voice assistant.

Case (number) name	(1) Jack	(2) Angela	(3) Jean	(4) Susan	(5) Peter
Sex	M	F	F	F	M
Age	92	87	87	89	95
Mobile phone	Y	Y	Y	Y	Y
Computer/tablet	Y	Y	Y	Y	N
Loneliness (pre-smart speaker)	All of the time	Sometimes	Sometimes	Rarely or none of the time	Rarely or none of the time

### Case 1 – Jack

Ninety-two-year-old Jack had recently moved into supported accommodation following the death of his wife. Jack reported at the start of the study that he was lonely all of the time, finding evenings particularly difficult when everyone would go to their room or apartment after dinner.

“I feel lonely most of the day because I lost my wife in March after 76 years. The most lonely time is in the evening, after our evening meal. Everything seems to shut up and we just go to our various rooms and either listen to the radio or watch television. I feel lonely, I am completely on my own.”

In terms of how he was spending his time at the start of the study, Jack went out on his motorized scooter to two local towns and saw his family once per week. Within the residential accommodation, he spent his afternoons playing Scrabble with other residents, watching television, or reading. Jack owned a mobile phone and tablet, which he used for emailing, and searching the internet for medical research, politics, and historical information.

At the end of the eight-week trial, Jack said he felt less lonely because of the smart speaker:

*“[it] keeps me company. I can talk to it, and it’s like another human being in the room.”* In terms of how he used the smart speaker, Jack listened to the radio and music, and asked general knowledge questions, for example, to help with crosswords. He also used the smart speaker to play games and set alarms and liked to listen to his music. He described how his routine had changed to include the hub which:

“wakes me up at half seven. I listen to Radio 4 in the morning to catch up with the news. In the early evening I use it when I’m doing my crossword and then I listen to Classic FM until bedtime.”

When asked about his feelings towards the smart speaker, Jack said:

“I feel very grateful for it. I think it is a wonderful thing because it brings another person into the room…I can listen to the radio, listen to any kind of music I like. I can ask it silly questions and it just keeps me company. It prevents loneliness…I can talk to it, and it just keeps me company, like another human being in the room.”

Jack found the smart speaker easy to use and very accessible:

“It’s easy. It knows my voice now, and there is no need to raise my voice. You do not have to get up to turn something on you just use your voice. Tell it to do something or ask a question and it comes with the answer. So, for a disabled person, it is even better, you can do everything you can do with a tablet, pretty well do everything if you are that way inclined. The trouble with old people these days is that they do not like using technology. They say they cannot do it, but they can. Nothing to turn on, the tuning or anything like that. You just ask it to do something like radio 4 or LBC, whatever you are listening to just use your voice.”

When asked if he would like to keep the device, Jack said:

“I would like to keep it permanently. I would be lost without it. If I didn't have it, it would be like losing somebody in my flat. I have company in my flat now with that, the [smart speaker], and without it, I would be lost…”

### Case 2 – Angela

Angela, an 87-year-old woman reported feeling lonely some of the time at the start of the study. She revealed that following the death of her husband, she had felt very lonely living in the flat they had shared as she “never saw anyone.” However, moving into supported accommodation had helped alleviate this to a certain extent. At the start of the study, she reported that she went out regularly, either walking or taking the bus into the local town. She also met with other residents for coffee and lunch and enjoyed occasional visits with her son. In the afternoons she watched television in her apartment. Angela used her mobile phone for communicating with her family and her computer for email and searching the internet, primarily to follow the news. When learning about the study Angela initially felt that Smart Speakers were perhaps a *“gimmick”.*

After using the smart speaker for eight weeks Angela reported that she found it “*filled the gap between television programs*.” In terms of how she used the device, Angela specifically used it to listen to music both on the radio and on Spotify. Angela said:

“I haven't done anything apart from music as I am quite happy with that. I get out but if I didn't get out, I would definitely want to hear a book, look at emails and look at the news, maybe learn a language… I would look things up.”

In terms of her daily routine, she reported since having the device:

“I get up and listen to the news and then I listen to music while I have my breakfast to cheer me up.”

Regarding the potential of smart speakers for tackling loneliness, Angela particularly remarked on the feelings listening to old, familiar music evoked:

“… I think these devices do help bring out certain feelings of nostalgia and satisfy nostalgia…it would go a long way to help isolation.”

At the end of the study, Angela purchased her own smart speaker.

### Case 3 – Jean

Jean was an 87-year-old woman who had moved into the supported housing from outside of the area due to mobility issues. She had been widowed for many years and did not have children. At the start of the study, she reported feeling lonely some of the time, but less so than before moving into supported accommodation. Jean particularly missed her earlier life when she ran a community art group with several companions, and it was the loss of this role and shared activity that made her feel lonely. To address this, she tried to go out every day but found that her mobility issues meant that she did not always make it. She used her mobile phone for calling taxis to take her out and speaking to her friends. Jean also used an iPad which she had owned “*for a very long time*,” of which she said:

“I couldn’t live without. It has all my friends on it, and I use it for everything. I email my friends and send them copies of my paintings.”

Over the eight weeks, Jean, like Angela, primarily used the smart speaker to listen to the radio and music. She particularly found that the ability to select whatever music took her fancy beneficial:

“It has changed my life in that I know I can listen to lovely music whenever I feel a bit lonely. When I am drawing or reading then the background music is very nice.”

Jean also reported another way that she used the smart speaker to lift her mood:

“I can ask it to tell me a joke every now and then. They are only children's jokes, but they are quite good. Every now and then I ask [it] to tell me a joke and it lightens the whole situation, that is very nice.”

Additionally, Jean used the smart speaker to listen to the news and had incorporated it into her daily routine by asking it to play music when she woke up in the morning. She reported an overall positive impact of the smart speaker:

“Having this device has changed my day, simply because it relaxes me and I am able to concentrate on other things and that is terribly good for the brain, at least for my brain, I don't know about other people. I would have thought it will be good for every brain.”

After using the smart speaker, Jean, like some of the other participants, reported that it created a positive presence for her. This supported her mental well-being and reduced her experience of loneliness:

“I always feel lonely, that is part of my personality. I think this device has changed my day simply because it makes me feel there is a presence in the room which is rather nice.”

Jean also commented that it was “*nice to command*” the device with her voice which she found particularly helpful. For example, she could control it from the patio without having to get up and go back inside, which she had to do for the radio. The control this gave her was very important for improving her life. Asked if she would like to keep the device, Jean said:

“I cannot think how I existed without it – I always have Spotify on.”

### Case 4 – Susan

Susan was an 89-year-old woman living with mobility problems, which contributed to her moving into supported housing. She had been a widow for 21 years but maintained strong family relationships, which were important in keeping any feelings of loneliness at bay. At the start of the study, she reported that she was unable to walk outdoors, so would take a taxi into the local town. She also reported being on good terms with most other residents with whom she had occasional chats, and spending time watching television, which she did not have time for before moving into supported accommodation. She always used her mobile phone, especially for text messages, and owned a tablet but reported that she did not know how to use it.

Susan found using the Smart Speaker *“very interesting and very useful.”* She mainly used it to find out information and check the weather. She particularly enjoyed asking questions and getting answers about sporting events and also used it to play games and set alarms.

“I enjoy using the device, simply because of what it does and the technology, which is quite remarkable and modern. I would say it is excellent and if anyone asked me about it, I would be encouraging them to have it.”

Although she did not feel lonely, she could see the potential for the smart speaker to help others, and maybe herself in the future…

“I do think it will be very helpful to some people for that purpose [loneliness]… there might come a time when I would need it more, if I was not in much contact with people.”

Finally, when asked how she would feel without it, Susan thought it would be difficult because she would want to find things out and would struggle to find answers without it. She added, *“I hope I’m not sounding greedy, but I would not like to be without it now.”*

### Case 5 – Peter

Ninety-five-year-old Peter had lived in supported housing for 4 years. Before moving into the supported housing, he had experienced loneliness following the death of his wife. Since moving, he reported the companionship at the supported housing had helped alleviate his loneliness. He supported a neighbour in the next-door room, by reading her letters and newspaper to her and changing batteries in her devices. He got on well with all of the residents and enjoyed shared dinners and conversations. At the start of the project, he went out every day to buy a newspaper and spent time sitting on his balcony “watching the world go by.” He also had regular visits with his son-in-law, who had adopted Peter’s dog, which he particularly enjoyed seeing again.

After using the smart speaker for eight weeks Peter described it as a *“miracle”*. He was amazed by what it could do…

“…I do the crossword in the newspaper… It comes up with the answers. It is all very helpful. General Knowledge. Now that I have had it and used it some time, I would not like to be without it. I just marvel at its ability to provide knowledge and answers. I enjoy it, wherever it gets its information from.”

Although he did not feel lonely, Peter found the interactive aspect also meant he had companionship, even feeling the device was *“becoming a friend”*…

“My life is different now because where in the past I was on my own in the room, and now I feel like I’ve got a companion. It’s nice to know that if you need to talk, it’s there and it’s like having a companion in the room that you talk to if you need to.”

Peter also saw that the smart speaker could be helpful for lonely people:

“It is comforting to think you have got a device that you can turn to if you need to. If you need to feel comfortable in the room without a companion that is the next best thing…if people do feel lonely and need companionship, beyond having someone sitting in the chair opposite you, it is nice to know that it is to hand and it would help. I am sure it would help somebody who did feel lonely. It would take some of the loneliness out of them.”

Like the other participants, Peter had incorporated the smart speaker into his daily life. At the end of the study he wanted to keep it, saying, *“It’s a wonderful thing and adds something to your life”*.

## Discussion

This community case study demonstrated the feasibility of introducing smart speakers to a small group of oldest old adults in a community setting and the ways in which these could provide a means of alleviating loneliness. The ease of using voice to control the device was a key factor in the successful commencement and adoption of the technology. These five older adults found that the smart speaker quickly became an indispensable item. As reported in other studies, they formed an attachment to their smart speakers which became embedded into their daily lives (Lopatovska and Williams, 2018; Ramadan et al., 2021). Convenience, including ease of use, along with the emotions they evoke and the identity they reinforce, is important for the experiential value older adults attribute to digital technologies (Desai et al., 2022). In the present study, these older adults all valued their smart speakers after eight weeks of use.

The impact of smart speakers on loneliness was attributed to two interrelated features: *presenc*e and *agency*. Presence was a tangible experience for the participants that mitigated loneliness and social isolation. As Jack reported: “*I have company in my flat now*,” suggesting that the oldest-old experience the same benefits of companionship as younger adults (O’Brien et al., 2020). Angela’s description of the smart speaker *“filling the gaps between television programs,”* suggested that for her it emulated a chat with a companion in the advertising breaks. Similarly, Peter, who did not feel lonely, reported that the device was “becoming a friend.” This echoes findings in previous studies where participants have formed digital relationships with smart speakers (Kim and Choudhury, 2021), particularly those who are lonely (Pradhan et al., 2019), who view them as companions (Corbett et al., 2021). Previous studies have identified a role for anthropomorphism in both companionship (Jones et al., 2021) and in mediating loneliness (Liu, 2023). In our small sample, the men treated the voice assistants as friends, which maybe a gendered issues to consider in future research where female voices are used.

Alongside presence, the oldest old participants in this study experienced *agency.* That is, the voice assistants gave them a means of not being alone. Essentially the availability of the smart speaker meant that older adults could choose not to be alone by hearing a voice or having an interaction with the device. Having the means to alter one’s situation is of major significance to people who are lonely and one that distinguishes smart speakers from interactions with telephones, computers, tablets or video calls. These latter modalities are used to connect to known contacts and are contingent on the other parties responding. People who are socially isolated by definition have very small social networks, which shrink further in later life (Clayton, 2018). Additionally, many older people report unequal social relationships, where they wait for family to contact or visit them (O’Neill et al., 2020). Being able to interact with the voice assistant whenever they choose, provides a new dimension to their experience of being alone.

In addition to creating an interactive presence for those who are alone, smart speakers are enjoyable and entertaining, fostering rather than hindering a sense of self and adding to the quality of life of these oldest-old adults. In this study, the participants reported that the smart speakers extended their interests and activities by providing access to new things. The three participants in this study who reported feeling lonely some or all of the time had experienced bereavement, along with feelings of loss and sadness resulting from changes to identity associated with being older (Clayton, 2018). Interacting with smart speakers, which filled the silence with music, jokes, and general knowledge, helped them cope with their loneliness.

The findings add to the growing body of evidence of how voice assistants can impact the experience of loneliness and social isolation. In addition to companionship, agency emerged in this study as a key factor in addressing loneliness and social isolation among these oldest-old adults. This has potential relevance for other socially isolated populations who have limited social networks. Additionally, these findings which confirm the ease of use of voice assistants can inform strategies to support implementation and adoption in housing and care settings for older adults and other populations who are digitally excluded (Holmes and Burgess, 2022).

## Limitations

Generalization of the findings of this pilot study is limited by the small sample size who were all current technology users and living in supported accommodation. Using a community setting and case study approach, however, facilitated the elicitation of individual experiences within a real-life community context (Baxter and Jack, 2008; Creswell, 2014). Whilst all five participants wished to keep the smart speakers, further research is required into their longer-term usage and benefits within supported accommodation and the wider community.

## Conclusion

In this community case study, smart speakers addressed loneliness among the oldest old adults, through providing a presence in their home. Voice control provided agency to these oldest-old adults to change their situation, an experience not afforded by other digital tools. Further research into the extent to which smart speakers can alleviate loneliness in the longer term is however necessary. Social care and health commissioners have not historically used this type of technology to address loneliness and the state of knowledge about the benefits of voice assistance is still emerging. This study contributes to building this evidence base.

A recent report for Vodafone (2019) highlighted that along with medical and social prescribing, there may be a case for “digital prescribing”. This is where pieces of technology may be purchased targeted at certain well-being outcomes like loneliness. As the older population is increasing and living longer, demand for this type of assistance is likely to increase. It is within this context, that smart speakers could be one such “digital prescription” as a device easy to procure, widely available, cheap, and programmable to help the oldest of old people cope with loneliness.

## Data availability statement

The raw data supporting the conclusions of this article will be made available by the authors, without undue reservation.

## Ethics statement

The studies involving humans were approved by University of Reading, Reading, UK. The studies were conducted in accordance with the local legislation and institutional requirements. The participants provided their written informed consent to participate in this study. Written informed consent was obtained from the individual(s) for the publication of any potentially identifiable images or data included in this article.

## Author contributions

AA: Conceptualization, Writing – original draft, Writing – review & editing. DC: Formal analysis, Writing – review & editing, Writing – original draft.

## Footnotes

^1^ https://www.thehumantech.agency/what/voiceforloneliness
